# Folding Mechanism of Beta-Hairpin Trpzip2: Heterogeneity, Transition State and Folding Pathways

**DOI:** 10.3390/ijms10062838

**Published:** 2009-06-22

**Authors:** Yi Xiao, Changjun Chen, Yi He

**Affiliations:** Biomolecular Physics and Modeling Group, Department of Physics, Huazhong University of Science and Technology, Wuhan 430074, Hubei, China; E-Mails: cjchen.hust@gmail.com (C.C.); brooksmiles@gmail.com (Y.H.)

**Keywords:** beta-hairpin, trpzip2, folding heterogeneity, transition state, folding pathway

## Abstract

We review the studies on the folding mechanism of the β-hairpin tryptophan zipper 2 (trpzip2) and present some additional computational results to refine the picture of folding heterogeneity and pathways. We show that trpzip2 can have a two-state or a multi-state folding pattern, depending on whether it folds within the native basin or through local state basins on the high-dimensional free energy surface; Trpzip2 can fold along different pathways according to the packing order of tryptophan pairs. We also point out some important problems related to the folding mechanism of trpzip2 that still need clarification, e.g., a wide distribution of the computed conformations for the transition state ensemble.

## 1. Introduction

The β-hairpin is one of the smallest protein building blocks that also has several qualities ascribed to proteins. Understanding the folding mechanisms of different β-hairpins can provide important insights into the protein folding problem, therefore, their folding thermodynamics and kinetics have been investigated extensively by experiments and theoretical simulations. In these studies one of the key aims has been to understand the mechanism of β-hairpin folding. These studies have provided many insights into their behaviors but also led to different proposed folding mechanisms.

One of extensively studied β-hairpins is the C-terminal of the immunoglobulin binding domain of streptococcal protein G (GB1). At least two main putative folding mechanisms have been proposed for GB1, including a zipper (zip-out) model [1,2] and a hydrophobic collapse model (zip-in and middle-out) [3,4]. The zipper model assumes that the folding of GB1 starts from the turn and propagates toward the tails by forming hydrogen bonds sequentially and the hydrophobic cluster forms later. This folding mechanism is supported by subsequent experiments and some simulations [5,6]. The hydrophobic collapse model assumes that the hydrophobic core forms first and then the structure propagates from there. This mechanism was supported by most simulation studies using different methods [7–12]. There is also a proposed reptation-like mechanism for β-hairpin folding, where a misregistered state with non-native backbone hydrogen bonds rearranges to the native structure by a sliding motion of the backbone along its length [13]. The reptation mechanism was first identified in simulation studies using a simplified model of the protein. Thus, we can see that there are different proposed folding mechanisms for GB1.

In this brief review we focus on another extensively studied b-hairpin: the tryptophan zipper 2 (trpzip2) [14]. The design of trpzip2 was based on the C-terminal of GB1 in order to build highly stable β-hairpin through introducing tryptophan-tryptophan cross-strand pairs. The folding kinetics and thermodynamics of the trpzip2 have also been extensively investigated both experimentally and computationally [6,14–24].

## 2. Folding Mechanism of Trpzip2

The 12-residue trpzip2 was synthesized as C-terminal amides by Cochran and coworkers [14]. It has a SWTWENGKWTWK sequence and adopts a unique tertiary fold without requiring special conditions such as metal binding, unusual amino acids, or disulfide crosslinks. Its native structure determined by NMR has an Asn-Gly turn and adopts a β-hairpin conformation with a type I’ β-turn at residues 6 and 7. The native β-hairpin structure has an obvious hydrophobic core composed of two aromatic side-chain pairs in edge-to-face pairwise interaction geometries, determined by electrostatic multipole moments [25]. The strong hydrophobic interactions of the two pairs of indole rings make the trpzip2 have significantly higher stability than most β-hairpins, even comparable to that of much larger protein domains. The thermal melting temperature *T*_m_ was found to be about 345K from the analysis of CD spectroscopy. The per-residue thermodynamic parameters indicate that the folding of the trpzip2, as in other proteins, is driven by burial of hydrophobic surface area. These make trpzip2 an ideal model for analysis of the thermodynamic and kinetic properties of β-hairpins. The trpzip2 has been also frequently used as benchmark to test the performance of new experimental techniques [26–30] and new or different computational methods [31–35]. In the following, we shall review the studies of the folding mechanism of the trpzip2 from three aspects: heterogeneity, transition state and folding pathways.

### 2.1. Folding Heterogeneity

Many proteins fold heterogeneously. Here the folding heterogeneity means that a protein can fold into the native state both rapidly in a two-state or even downhill manner with no apparent intermediates and more slowly in a multistate way in ensemble experiments. They occur with comparable probability and are observable. The free energy landscape of most protein folding is usually very rough and has many local stable states, which may become intermediates along the folding pathways. This may lead to heterogeneous folding because different intermediates may get populated or stabilized. It is interesting that the folding of the small peptide trpzip2 is also found to be heterogeneous.

The first experimental and computational studies of the folding heterogeneity of the trpzip2 appeared in 2004. Yang *et al*. [16] presented experimental (UV CD and IR) thermodynamic replica exchange molecular dynamics (REMD) studies on trpzip2. Their experimental data showed three separate transitions in the equilibrium thermal unfolding process: (1) a transition at low temperature (15–40 °C) caused by loosening of the tryptophan side chains; (2) a main transition near 60–70 °C with significant populated partially folded populations, e.g., different pair clustering of the four tryptophan residues; (3) a final transition that may involves breaking up residual random contacts between the tryptophans, further extending the global compactness of the peptide. Their simulations also indicated that trpzip2 has three separate transition regions (17–32 °C, 48–75 °C and 162 °C) when monitored as a function of temperature by different molecular dynamics simulation observables (Cα-RMSD and tryptophan χ1, number of backbone hydrogen bonds and fraction of terminal salt bridges, radius of gyration). Furthermore, these results were confirmed by their experiments using fluorescence spectroscopy of different wavelengths [17] and later by the simulations of Wang’s group [21], although Wang’s group found two transitions instead of three in the unfolding process: the first melting transition spans a temperature range from 362–370 K (89–97 °C), while the second is at about 392 K (119 °C). Both groups simulated the unfolding of the trpzip2 using all-atom REMD but with different solvent models. This suggests that the discrepancy may be due to the different water models used in the two simulations. It is also possible that one or more of these simulations might not have been run long enough to converge.

On the other hand, there are also experiments and simulations that show a two-state folding pattern for trpzip2 at the normal temperature. Pande’s group [18] found that the free-energy landscape for trpzip2 folding does not contain large free energy traps hindering folding. The folding activation enthalpies are 4–7 KJ/mol (simulation), 15.9±0.3 KJ/mol (fluorescence) and 17.9±1.1 KJ/mol (IR), in contrast to the unfolding activation enthalpies (53–57 KJ/mol - simulation, 50.2±0.6 KJ/mol - fluorescence and 73.7±1.4 KJ/mol - IR). Yang *et al*. [36] simulated trpzip2 at 300K and this gave both rough and smooth free energy profiles, depending on reaction coordinates. It is noted that the two-state folding pattern of trpzip2 can also occur at high temperature. For example, Gruebele and coworkers showed that, when the simulation temperature is high enough, the calculated free energy profile shows a two-state pattern. Ulmschneider and coworker [19,20] obtained the similar result by both Monte Carlo (MC) and MD simulations at 323K, which is near the melting temperature of 345 K.

Trpzip2 has four tryptophan residues and so it is expected that the multiple tryptophan interactions may lead to different nonnative hydrophobic cores. These nonnative hydrophobic cores are very stable and make the free energy landscape very rough. Gruebele and coworkers [16] found that the local minima (other than the native state) of the trpzip2 free energy landscape range from 1 to 4 kT at 310K. They later also estimated the roughness (about 0.6k^2^T^2^) of trpzip2 free energy landscape directly from experiments, which agreed well with the MD simulation [17]. Their simulations revealed at least seven structurally distinct local minima at low temperatures. These local minima correspond to fairly compact structures, with distinct patterns of tryptophan-tryptophan interactions. Later, several research groups obtained similar results. Wang and coworkers [21] identified six basins of attractions on the free energy surface: the native state (N), the unfolded state (U), a compact state H (including H1 and H2), a nearly folded state P, and a possibly misfolded state M, where the H1 and H2 states have different packed nonnative hydrophobic cores. Pitera *et al*. [22,37] found some misregistered states with significant populations.

Our recent simulations [24] refine the picture of trpzip2 folding heterogeneity. We have done thirty-eight 100-ns all-atom molecular dynamics simulations at 298K starting from an extended sate. These 38 trajectories are independent and continuous. We find seven basins in the free energy landscape, which include a native (containing the native state) and a nearly native basin, three hairpin-like and an asymmetric hairpin basins with different nonnative packing of three tryptophan residues, and a basin with one or more helix turns. Along the Cα-RMSD reaction coordinates, the barriers between the non-native basins are about 0.2–0.6 kcal/mol and that between the native and non-native basins is about 0.9 kcal/mol. This confirms the roughness of the free energy landscape of trpzip2 at normal temperatures.

There are two different interpretations of the heterogeneous protein folding kinetics. One is in terms of multiple independent unrelated pathways and another is in terms of a predetermined pathway with different probabilities staying in the on-pathway intermediates [16,38]. Our recent folding network analysis of the simulated trajectories given in ref. [24] reveals that trpzip2 can have two-state or multi-state folding, depending on whether it folds within the native basin or through the non-native basins. Each basin in the free energy surface is like a smooth funnel and between them there are high free-energy barriers. Therefore, if the peptide folds only within the native basin, it folds into the native state very quickly in a two-state or even downhill manner. However, if it folds through one or more non-native basins except the native basin, it will overcome the barriers in order to fold into the native state and shows a multi-state folding. In fact, among the 38 simulated trajectories, eight trajectories fold within the native basin and into the native state in a few ns or even less than 1 ns. Two trajectories fold through more than two non-native basins and into the native state in more than 50 ns and show a multi-state pattern. Another 28 trajectories don’t fold into the native state and are trapped in the non-native basins. We needed longer simulation times to fold them into the native state and they will also show multi-state folding. Thus, the fast and slow folding occurs with probabilities of about 21% and 79% respectively and both should be observable in experiments. In fact, the experimental data obtained by Yang *et al*. [16] showed three separate transitions in the equilibrium thermal unfolding process while that of Pande’s group [18] seems to suggests a two-state folding. Therefore, the folding heterogeneity of trpzip2 may be caused by both multiple independent pathways and different probabilities staying in the non-native basins.

### 2.2. Transition State

By definition the transition state is the most unstable state (the free energy maximum or separatrix) along a folding pathway and has equal probability of relaxing to the native state and denatured state [39]. Determining transition state conformations is crucial to identifying protein folding pathways and the key residues that determine folding dynamics. The transition-state conformations cannot be observed directly in experiments. Therefore, molecular dynamics simulations provide a useful tool to study the conformations of the transition states of protein folding.

Many groups have investigated the conformations of the transition state of trpzip2 folding by molecular dynamics simulations, but obtained very different results. The transition state ensemble contains a wide distribution of conformations. It may be argued that this is due to different folding pathways. However, even for the zip-out pathway, there are three types of calculated conformations for the transition state:

1. The transition state is similar to the native state. For example, Pande’s group [18] found the transition state is similar to the native state except the tryptophan-2 having large fluctuations. Xu *et al*. [23] found that the transition state ensemble is characterized by a largely formed turn and a compact packing of tryptophanes 2, 4 and 9.
2. The transition state is characterized by the formation of the inner hydrogen bonds and the inner pair of hydrophobic residues. For example, Wang’s group [21] found that the transition state is characterized by a largely formed turn, high probabilities of the three inner hydrogen bonds, and the hydrophobic interaction between the inner pair of core residues. Similarly, our unpublished studies of the folding processes of trpzip2 by the united residue (UNRES) model [40] identified that the transition state conformation is characterized by the inner one or two native contacts formed and two tails still having a larger distance.
3. The transition state is characterized by the formation of only inner native hydrogen bonds. For example, Yang *et al*. [36] identified the two types of transition state for trpzip2. One has a conformation with two inner native hydrogen bonds being formed (zip-out pathway). Another has a conformation with two outer native hydrogen bonds being formed (zip-in pathway). There is also a related example: Gai and coworkers [6] compared the folding and unfolding kinetics of trpzip4 and the GB1 β-hairpin differing from each other only in the composition of the hydrophobic cluster and showed that these two β-hairpins have similar folding rate but significantly different unfolding rates. Furthermore, due to the triple tryptophan mutations in the GB1 β-hairpin, the φ value of trpzip4 for folding is about 0.11, suggesting that the two native hydrophobic pairs have not been formed in the transition state.

One of the main reasons for the wide distribution of transition state conformations may be the different methods used to identify the transition state. Pande’s group [18] characterized the transition state by identifying conformations that will fold within the next 250 ps. They used 250 ps here because their trajectory conformations were recorded in this time interval. It is clear that the transition state defined by this way is a “lowest” limit with respect to the native state, i.e., under the assumption that commitment to the unfolded or folded states is fast (shorter than 250 ps), this method should identify members of a putative transition state ensemble. This may be the reason why it is close to the native state. Wang’s group [21] identified the structures of the transition state using a so-called “FF process” [41] and defined the transition region as the energy barrier separating the native and denatured basins on the projected free energy surfaces. Yang *et al*. [36] also defined the transition region based on the projected free energy surfaces and identified the transition states by searching the snapshots from the simulated trajectories. Both works used the projected free energy surfaces to define the transition region. However, since the projected free energy surface is just a two-dimensional reduction of the real high-dimensional free energy surface, whether the determined transition region is really the correct one need to be further proved. Therefore, none of the methods here are guaranteed to identify configurations on the separatrix and we need a more reliable method to do this.

### 2.3. Folding Pathways

One of the aims of studying mechanism of protein folding is to understand how different types of interactions determine folding pathways. Here the folding pathway is referred to as the process by which a completely disordered peptide (with no intrachain contacts or hydrogen bonds) folds into the NMR-resolved hairpin structure. The folding pathways of beta-hairpin peptide, especially GB1 and trpzip2, have been intensively investigated since the zipper (zip-out) model [1,2] and the hydrophobic collapse model (zip-in and middle-out) [3,4] were proposed. Here the main problem is the order of formation of the native hydrogen bonds and the hydrophobic core, the tryptophan pairs in particular for trpzip2. In fact this concerns the role of the formation of the native hydrophobic core in hairpin formation. For GB1, there are two different points of view: the turn forming first and the hydrophobic core forming later or the inverse. The former implies that the hydrophobic interactions may not play a leading role in the correct formation of the native hairpin structure of GB1. For trpzip2, all the simulations suggest that zip-out is the main folding pathway. However, other pathways are also possible and can have significant probability of occurrence (see the following). This suggests the trpzip2 may have multiple folding pathways. Furthermore, the simulations also show that the ways of hydrophobic core formation play important roles in determining which folding pathway to go along.

Experimentally, there are no direct demonstrations of how trpzip2 folds. However, there are a few related experiments. Gai and coworkers [6] investigated the key factors that determine the folding and unfolding rates of tryptophan zippers by using T-jump IR and fluorescence. They systematically analyzed the folding kinetics of four trpzips (including trpzip2), which differ only in the turn sequence. They found, although the unfolding rate constants of these trpzips are different only by a factor of three or less, their folding rate constants differ by as much as 30-fold. This result suggests that the turn sequence is a strong determinant of the trpzip folding rate but has a rather small effect on the unfolding rate. This supports a folding mechanism with the rate-limiting event corresponds to the formation of the turn. They considered this is similar to a zipper model conceptually. However, Smith *et al*. [42] investigated the thermal denaturation of trpzip2 between 15 °C and 82 °C using two-dimensional IR vibrational spectroscopy, dispersed vibrational echo spectroscopy, and Fourier transform IR spectroscopy. They found the interstrand coupling between local amide I oscillators along the peptide backbone at all temperatures. This indicates that stable hydrogen bond interactions persist between the two β-strands in the thermally denatured state of trpzip2, so their formation is likely not relevant to the relaxation behavior observed in a T-jump kinetics experiment that jumps across T_m_ and watches relaxation of tryptophan fluorescence of FTIR. Therefore, further experiments are needed to determine whether the turn formation as the rate-limiting event is similar to a zipper model.

As mentioned, computationally, almost all simulations suggest that zip-out is the main folding pathway of trpzip2. However, other pathways are also possible. Pande’s group [18] found that for trpzip2, on average, the inner tryptophan pair and inner hydrogen bonds generally formed first, and formation of the final hydrogen bonds usually occurred with correct tryptophan packing. Wang’s group [21] and Yang *et al*. [36] showed that the folding of trpzip2 is mainly a zip-out process, but there are also a small number of zip-in trajectories. Xu *et al*. [23] found that the outer tryptophan pair forms at a very late stage of the folding process, the first (closest to the turn) and the fourth native hydrogen bonds form earlier, but they did not observe a strict zip-out process of hydrogen bond formation. In addition, Pitera *et al*. [22] carried out high-temperature MD simulations of the trpzip2 to find evidence of reptation folding process, but they didn’t observe any evidence of this. The misregistered states can go to the native state only by passing through the unfolding states via significant expansion of the polypeptide chain.

Our recent large scale simulations of all-atom model [24] and UNRES model (unpublished) give a more complete picture of the trpzip2 folding pathways. We not only confirmed that the zip-out is the most probable folding pathway for trpzip2, but also found three other pathways: zip-in, middle-out and fast non-zipper. The zip-in pathway has also been mentioned by other works [21,36], but this represents the first observation of the middle-out and fast non-zipper pathways in trpzip2 folding. In the non-zipper pathway the native hydrophobic core and hydrogen bonds form simultaneously and the trpzip2 folds into its native state very fast (within 0.2 ns in our simulations). Furthermore, although the zip-in and middle-out pathways occur with lower probabilities (10%, respectively, in all-atom simulations), the fast non-zipper pathway occurs with a large probability (30% among ten folded events in all atom simulation and 28% among 374 folded events in UNRES simulation) and may be observed by ultrafast measurement. This indicates that at least there are two observable pathways for beta-hairpin folding.

Although all the simulations show a favorable zip-out pathway or the turn forming first, some of them also indicated the role of the formation of the native hydrophobic core in the turn and hydrogen-bond formations. For example, Wang’s group [21] found that the turn formation and establishment of hydrophobic interactions are cooperative processes and occur almost simultaneously. They suggested a folding picture of a blend of hydrogen bond-centric and hydrophobic core-centric mechanism. Our simulations [24] further show that which pathway the trpzip2 folds along depends on the ways how the two hydrophobic pairs approach to their native conformations, which is consistent with the earlier observations in a lattice model by Imamura and Chen [43]. If the inner hydrophobic pair firstly forms its native conformation, the peptide folds along a zip-out pathway. If the outer hydrophobic pair firstly forms its native conformation, the peptide folds along a “zip-in” pathway. If the two hydrophobic pairs form their native conformations simultaneously, the peptide folds along a “middle-out” pathway. Furthermore, there is a non-zipper pathway if two hydrophobic pairs and five native hydrogen bonds forming simultaneously. These results indicate that all the folding pathways, whenever zip-out (the turn forming first), zip-in or middle-out, are related to the correct formations of the native hydrophobic core or partial native hydrophobic cores. This is in agreement with the generally accepted view that the main driving force of protein folding is the hydrophobic one.

## 3. Conclusions

In summary, from these studies of the trpzip2, we can conclude the following: (1) The free energy landscape of the trpzip2 folding and unfolding is very rough at normal temperatures due to the multiple tryptophan interactions. There are a number of stable intermediate states located on the free energy surface. The transitions between them make the folding and unfolding processes of trpzip2 heterogeneous and not necessarily a simple two-state ones. The heterogeneity of trpzip2 folding may be caused by both multiple independent pathways and different probabilities staying in the non-native basins. (2) Most studies have found that the zip-out is the main folding pathway of the trpzip2 but there exist other possible folding pathways. All proposed possible folding pathways (zip-out, zip-in and middle-out) for β-hairpin have been observed computationally in trpzip2 folding except reptation. There also exists a fast folding pathway that should be observable by experiment. Furthermore, the hydrophobic interactions play important roles in all these folding pathways and different packing orders may lead to different folding pathways. This may be a manifestation of how an amino acid sequence determines its folding pathway. However, a large-scale of all-atom molecular dynamics simulations is needed to get a more clear and detailed picture, including the identification of reptation mechanism. (3) There exists a wide distribution of conformations of the transition state. Even for the zip-out pathway, there are three types of conformations of the transition state: one kind of the identified conformations of the transition state is similar to the native state; one is characterized by the formation of the inner hydrogen bonds and the inner pair of hydrophobic residues; one is by the formation of only inner native hydrogen bonds. This may be due to different methods to characterize or extract the transition state. Therefore, more accurate method is needed to characterize the transition state. The existed simulation studies also show that the AMBER96 force field plus the GB/SA implicit solvent model or the explicit TIP3P water model may be reasonable to simulate the folding dynamics of the trpzip2. The existed studies reviewed above have given deep insight into the folding mechanism of the trpzip2. However, more detailed investigations are needed to clarify the mentioned inconsistencies and give a complete picture.
